# Deletion of Chromosomal Region 8p21 Confers Resistance to Bortezomib and Is Associated with Upregulated Decoy TRAIL Receptor Expression in Patients with Multiple Myeloma

**DOI:** 10.1371/journal.pone.0138248

**Published:** 2015-09-17

**Authors:** Adil Doganay Duru, Tolga Sutlu, Ann Wallblom, Katarina Uttervall, Johan Lund, Birgitta Stellan, Gösta Gahrton, Hareth Nahi, Evren Alici

**Affiliations:** 1 Center for Hematology and Regenerative Medicine, Karolinska Institutet, Karolinska University Hospital Huddinge, Stockholm, Sweden; 2 Center for Diseases of Aging, Vaccine and Gene Therapy Institute of Florida, Port St. Lucie, Florida, United States of America; 3 Nanotechnology Research and Application Center, Sabanci University, Istanbul, Turkey; 4 Haematology Centre, Karolinska University Hospital, Huddinge, Stockholm, Sweden; Virginia Commonwealth University, UNITED STATES

## Abstract

Loss of the chromosomal region 8p21 negatively effects survival in patients with multiple myeloma (MM) that undergo autologous stem cell transplantation (ASCT). In this study, we aimed to identify the immunological and molecular consequences of del(8)(p21) with regards to treatment response and bortezomib resistance.

In patients receiving bortezomib as a single first line agent without any high-dose therapy, we have observed that patients with del(8)(p21) responded poorly to bortezomib with 50% showing no response while patients without the deletion had a response rate of 90%. *In vitro* analysis revealed a higher resistance to bortezomib possibly due to an altered gene expression profile caused by del(8)(p21) including genes such as TRAIL-R4, CCDC25, RHOBTB2, PTK2B, SCARA3, MYC, BCL2 and TP53. Furthermore, while bortezomib sensitized MM cells without del(8)(p21) to TRAIL/APO2L mediated apoptosis, in cells with del(8)(p21) bortezomib failed to upregulate the pro-apoptotic death receptors TRAIL-R1 and TRAIL-R2 which are located on the 8p21 region. Also expressing higher levels of the decoy death receptor TRAIL-R4, these cells were largely resistant to TRAIL/APO2L mediated apoptosis.

Corroborating the clinical outcome of the patients, our data provides a potential explanation regarding the poor response of MM patients with del(8)(p21) to bortezomib treatment. Furthermore, our clinical analysis suggests that including immunomodulatory agents such as Lenalidomide in the treatment regimen may help to overcome this negative effect, providing an alternative consideration in treatment planning of MM patients with del(8)(p21).

## Introduction

Multiple Myeloma (MM) is a malignant neoplasm that accounts for about 20% of deaths caused by hematological malignancies and is characterized by clonal proliferation of plasma cells in the bone marrow (BM). The current gold standard for the treatment of patients under 65 years of age is high-dose chemotherapy (HDT) followed by autologous stem cell transplantation (ASCT)[1]. In the last decade, the addition of novel agents such as the proteasome inhibitor bortezomib (Velcade ®) to the regimen has resulted in a substantial increase in the number of patients responding to therapy[2]. Following these results, bortezomib is included in almost all the treatment regimens in the 1^st^ line treatment of MM patients and is currently considered to be the backbone in modern treatment of MM patients. Yet, approximately 20% of patients do not respond to bortezomib[3]. Defining a mechanism of drug resistance in these patients may have a direct implication on the choice of treatment modality.

While bortezomib exerts its major activity by inhibiting the chymotrypsin-like proteolytic activity of the 26S proteasome and promoting the accumulation of inefficiently degraded proteins leading to apoptosis, several studies have also shown that it is a key player in sensitization of MM cells to apoptosis induced by TRAIL/APO2L via upregulation of TRAIL receptors 1 and 2[4].

We have recently discovered that loss of the chromosomal region 8p21 is an independent prognostic factor associated with poor survival in MM patients receiving standard ASCT[5, 6]. These findings have been confirmed by other groups who have reported similar results[7, 8]. The tumor necrosis factor-related apoptosis inducing ligand (TRAIL) receptor gene cluster as well as several other genes such as PTK2B[9] and SCARA3[10] that might have a role in multiple myeloma progression and treatment resistance, lie in the *p* arm of chromosome 8. However, the effect of the deletion on the bortezomib resistance and bortezomib mediated sensitization to TRAIL/APO2L killing has been left as a speculation so far. While the alteration of cell surface TRAIL receptor expression due to del(8)(p21) might cause decreased sensitivity of tumor cells to TRAIL-mediated apoptosis[11] [12], it must be noted that these clones still carry one copy of each TRAIL-R gene, since the deletion in the 8p21 region is almost exclusively monoallelic. Therefore, bortezomib treatment might still upregulate TRAIL receptor expression and break immune tolerance resulting in efficient elimination of MM cells with 8p21 deletion.

In this study, in order to identify the consequences of del(8)(p21), with a special focus on TRAIL/APO2L mediated killing, we have analyzed the expression of various genes on the 8p21 region as well as others in patients with and without del(8)(p21). Additionally, we have analyzed the response of MM cells with and without the deletion to bortezomib mediated killing and sensitization to TRAIL/APO2L triggered apoptosis in an attempt to understand why MM patients carrying 8p21 deletion respond poorly to bortezomib treatment.

## Materials and Methods

### Patient material and cell lines

The patients were admitted to the Department of Medicine, Karolinska University Hospital Huddinge, Stockholm, Sweden. This study was specifically approved by the local research ethics committee (Etikprövningsnämnden Stockholm, Ethical permit number: 2014/526-31). Written informed consent was obtained from all patients in accordance with the Declaration of Helsinki and with the local ethical committee guidelines.

Bone marrow aspirates at diagnosis were separated by density gradient centrifugation (Lymphoprep^TM^, Axis-Shield, Oslo, Norway). Plasma cells were positively selected by CD138 magnetic beads using AutoMACS (Miltenyi Biotec, Bergisch Gladbach, Germany). K562 and U266 cells were maintained in RPMI 1640 medium (GIBCO^®^, Life Technologies, Carlsbad, CA) supplemented with 10% FBS (GIBCO).

### Analysis of mRNA levels

A TaqMan^®^ array micro fluidic card (TLDA, Applied Biosystems^®^, Life Technologies) was designed in order to study the expression of a selected set of genes on the 8p21 region as well as some additional genes that were reported to be overexpressed in MM[13] and had functional links to genes located on the 8p21 region. The full list of genes and TaqMan^®^ assay IDs can be found in S1 Table.

Total RNA was extracted from TRIZOL^®^ (Life Technologies) treated CD138^+^ magnetically separated bone marrow cells and RNA was isolated using RNeasy Plus Mini Kit (Qiagen, Limburg, the Netherlands) according to manufacturer’s protocol. 800 ng of total RNA was used for cDNA synthesis using Superscript III (Invitrogen) according to manufacturer’s protocol. The reaction mixture containing 50 μl cDNA template (200 ng) and an equal volume of TaqMan^®^ universal master mix (Applied Biosystems) was added to each line of TLDA after gentle vortex mixing. The samples were run as triplicates on a 79000HT Fast-Real-Time PCR System (Applied Biosystems) and the cycling conditions were as follows: 2 min at 50°C, 10 min at 94.5°C and 30 s at 97°C, and 1 min at 59.7°C for 40 cycles.

Analysis was done using DataAssist software (Life Technologies) and threshold cycle Ct was automatically determined. The expression of GAPDH and ACTB genes were used as endogenous control genes while RNA samples from K562 and U266 cell lines were used as calibrator samples to analyze relative gene expression in the patient samples. Data derived from the TLDA were normalized against the average values of the control genes. Relative quantities (RQ) were determined using the equation: RQ = 2^-ddCt^.

### Analysis of TRAIL receptor cell surface expression

CD138^+^ plasma cells or CD138^-^ bone marrow mononuclear cells (BM-MNC) were cultured in RPMI 1640 medium supplemented with 10% FBS in the presence or absence of 10 nM bortezomib for 24 hours in U-bottom 96-well plates. Cells were washed with PBS and stained with fluorescently labeled anti-TRAIL receptor antibodies, TRAIL-R1 (DR-4-02), TRAIL-R2 (DR5-01-1), TRAIL-R3 (TRAIL-R2-02) and TRAIL-R4 (TRAIL-R4-01) (Thermo Scientific); anti-CD38 (HIT2, BD Biosciences, San Jose, CA) and anti-CD138 (MI15, BD Biosciences), additionally CD138^-^ BM-MNC were stained with anti-CD45 (HI30, BD Biosciences) antibody 30 minutes on ice. The cells were then washed and resuspended in PBS at 4°C. Relative expression of each TRAIL receptor was normalized by MFI of corresponding isotype control.

### Analysis of apoptosis upon bortezomib and/or soluble TRAIL treatment

CD138^+^ cells were cultured in RPMI with 10% FBS overnight, together with or without 10 nM bortezomib followed by a 6 hour incubation with 250 ng/ml soluble TRAIL/APO2L. The cells were stained with anti-CD138 and anti-CD38 antibodies for 30 minutes on ice followed by Annexin V and propidium iodide (PI) staining according to manufacturer’s instructions (BD Biosciences). Percentage of Annexin V^-^PI^-^CD38^+^CD138^+^ plasma cells in samples that were not incubated with bortezomib and TRAIL/APO2L were used as controls. All flow cytometry data was acquired using CyFlow ML (Partec, Munster, Germany) and analyzed by Flowjo software (Tree Star, Ashland, OR, USA).

### Analysis of chromosomal abnormalities by fluorescent in situ hybridization (FISH)

CD138^+^ plasma cells (2–4 x 10^4^ cells/spot) were centrifuged to prepare hybridization slides using CytoSpin (Thermo Scientific, Pittsburg, PA), air-dried overnight and stored at -20°C. For FISH analysis, all cases were investigated with *in vitro* diagnosis certified probe sets targeting 1q21/8p21, 6q21/15q22, 17p13.1/19q13, 9p21/9q21, 13q14/qter, and for translocations t(4;14)(p16.3;q32.3), t(11;14)(q13;q32.3), t(14;16)(q32.3;q23) (Kreatech, Amsterdam, the Netherlands). Hybridization and detection of signals were performed according to the manufacturer’s protocols. Analysis of spot counting was done in Olympus microscope (BX60, Tokyo, Japan). 200 nuclei were evaluated for each probe. Full list of patient specific chromosomal abnormalities observed in this study is listed in S3 and S4 Tables.

### Patients and treatment

140 consecutive patients diagnosed with MM at our hospital between 2008 until the end of December 2012 were included of which 37 patients (26%) had del(8)(p21). Patients with plasma cell leukemia or solitary plasmacytoma were excluded. Eighty eight of the 140 patients were treated with bortezomib in 1^st^ line, 24 with del(8)(p21) and 64 without. Bortezomib based induction was given as VCD (bortezomib/Cyclophosphamide/Dexamethasone) and followed by conditioning with melphalan (200 mg/m^2^) to all HDT patients. The non-HDT population received either VCD (69%) or VMP (bortezomib/Melphalan/Dexamethasone) (31%). 34 patients were treated in 2^nd^ line with Lenalidomide and Dexamethasone; 16 with prior bortezomib treatment in 1^st^ line and no HDT and 18 with VCD induction and HDT. Response to treatment was evaluated according to Blade *et al* [14].

### Statistical Analysis

**Fig 1:**
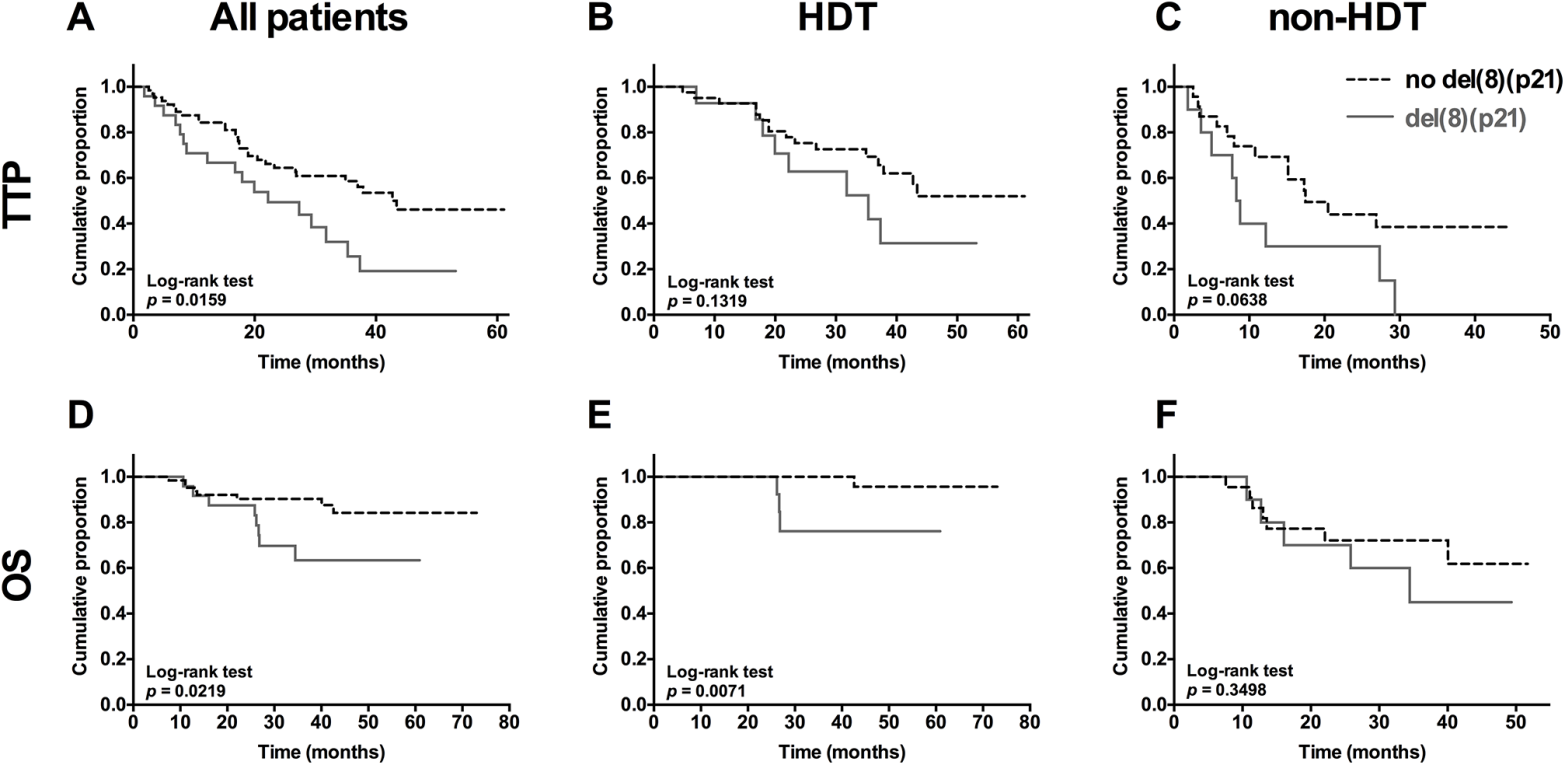
Multiple myeloma patients with del(8)(p21) have poor TTP and OS. Time to progression (TTP) of (A) all patients, (B) HDT patients, (C) non-HDT patients after diagnosis (months). Overall survival (OS) of (D) all patients, (E) HDT patients, (F) non-HDT patients after diagnosis (months). Patients with del(8)(p21) (n = 24). Patients without del(8)(p21) (n = 64). HDT patients with del(8)(p21) (n = 14). HDT patients without del(8)(p21) (n = 41). Non-HDT patients with del(8)(p21) (n = 10). Non-HDT patients without del(8)(p21) (n = 23). Log-rank (Mantel-cox) test performed using Graphpad Prism software.

**Fig 2:**
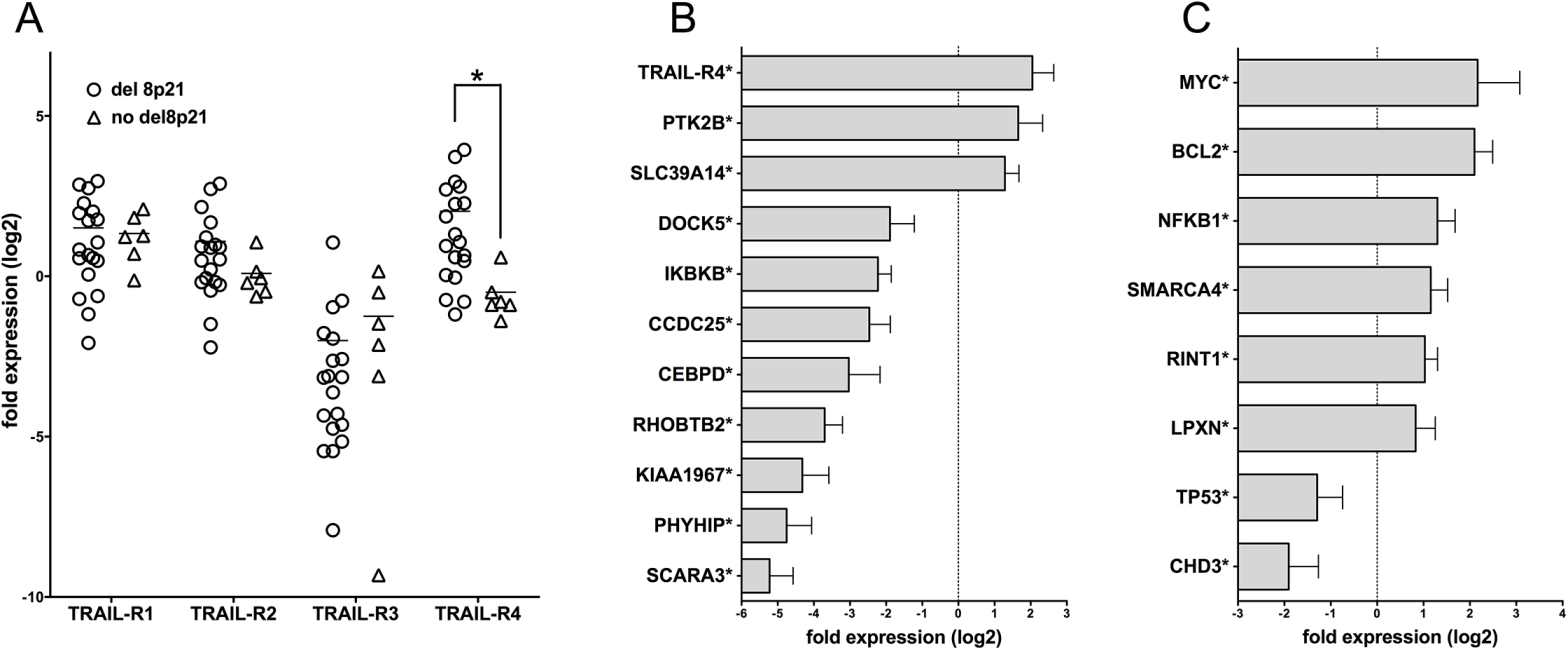
Quantitative RT-PCR analysis of gene expression in MM patients with or without del(8)(p21). TLDA cards were used to analyze mRNA levels in patients with (n = 19) and without (n = 6) del(8)(p21). For the analysis, GAPDH and ACTB genes were used as endogenous controls while RNA samples from K562 and U266 cell lines were used as calibrators. (*p<0.05, unpaired t test) (A) The mRNA level relative expression of TRAIL receptors in patients with and without the deletion. TRAIL-R1,-R2 and–R3 did not show any change while TRAIL-R4 was expressed at significantly higher levels in patients carrying the deletion. (B) Genes on or near 8p21 **(C)** other analyzed genes located elsewhere in the chromosome that show at least two-fold differential expression in MM cells with the deletion. Depicted are the gene expression levels in MM cells carrying the deletion, normalized to samples without the deletion.

**Fig 3:**
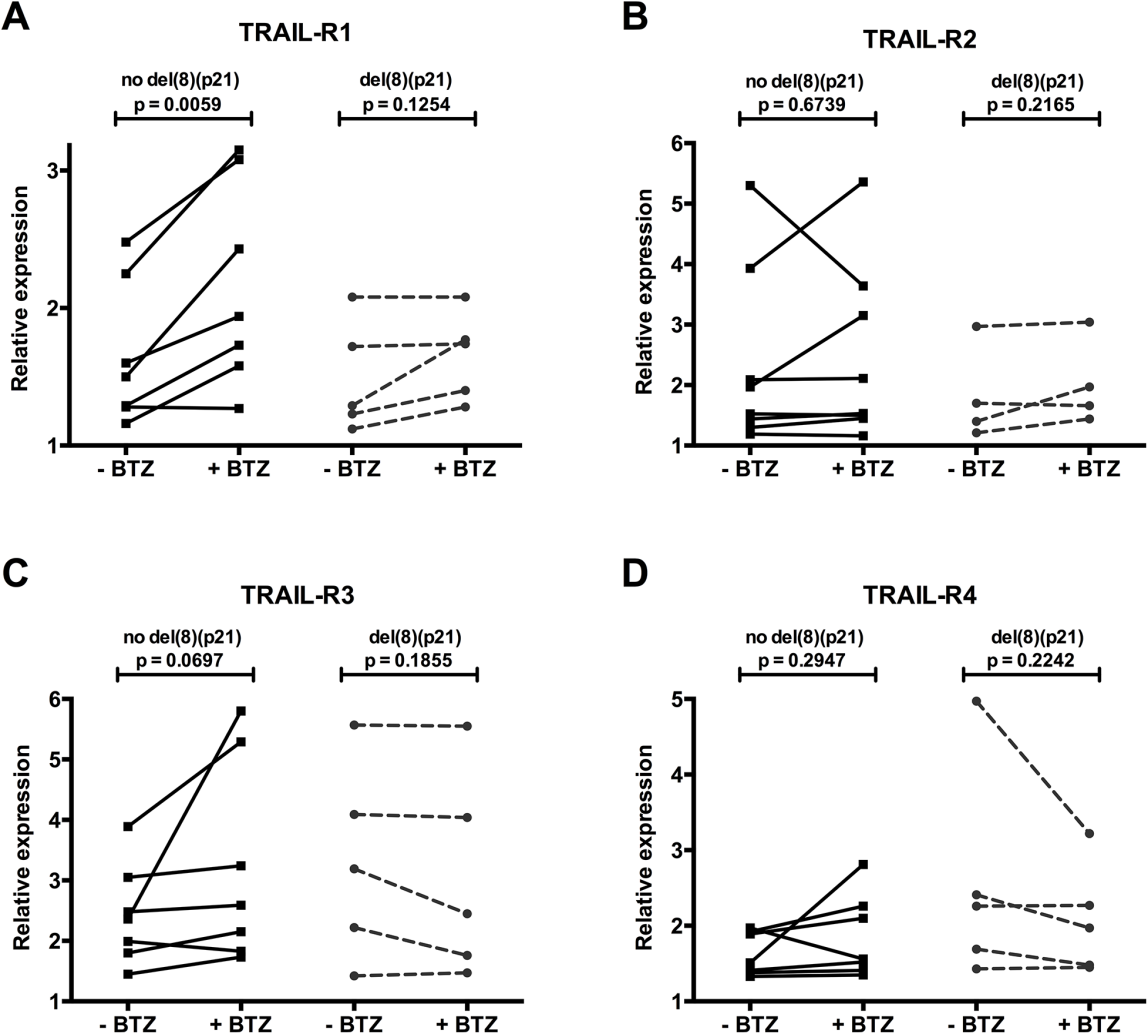
Patients with del(8)(p21) fail to upregulate pro-apoptotic TRAIL receptor expression upon bortezomib treatment. Relative expression of TRAIL receptors of 5 MM patients with del(8)(p21) and 7 MM patients without deletion is determined by flow cytometry. Cell surface Expression levels are normalized to corresponding isotype controls. **(A)** TRAIL-R1, **(B)** TRAIL-R2, **(C)** TRAIL-R3, **(D)** TRAIL-R4. Effect of bortezomib treatment on TRAIL receptor expression is analyzed by paired t-test.

**Fig 4:**
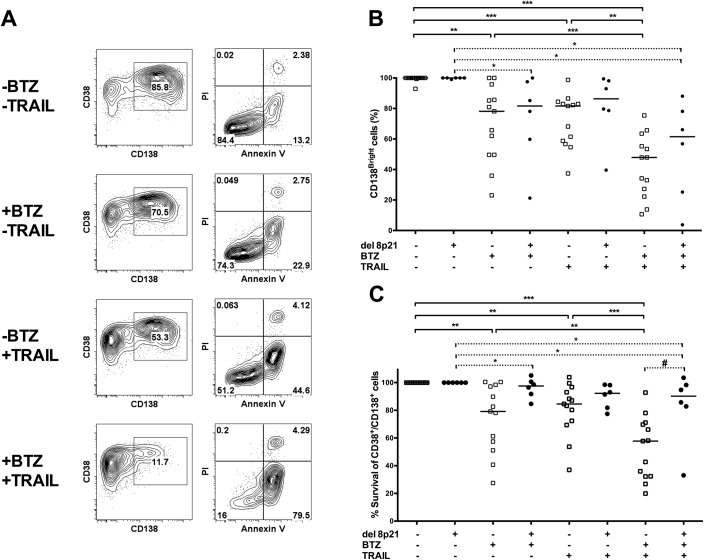
Patients with del(8)(p21) are less sensitive to bortezomib and TRAIL mediated apoptosis. CD138 expression levels and Annexin V/PI staining was performed to assess viable, apoptotic and dead cells. Mononuclear cells were separated by Lymphoprep from fresh bone marrow aspirates of MM patients followed by CD138 positive magnetic selection. Cells were incubated overnight with or without 10 nM bortezomib followed by incubation with or without 250 ng/ml soluble TRAIL/APO2L for 6 hours. Thereafter, cells were stained with anti-CD38, anti-CD138, anti-Annexin V antibodies and PI. (A) Representative patient data displaying relative changes in CD38^+^/CD138^Bright^ cell population (left panel) and Annexin V/PI (right panel) upon bortezomib and/or soluble TRAIL/APO2L treatment. (B) Relative changes in the CD38^+^/CD138^Bright^ cell population of 13 MM patients without del(8)(p21) and 6 MM patients with del(8)(p21) upon bortezomib and/or soluble TRAIL/APO2L treatment is displayed. Amount of CD38^+^/CD138^Bright^ cells of each untreated patient MM cells is considered as 100%. (C) Relative percentage of live MM cells (Annexin V^-^ and PI^-^ cells) of 13 MM patients without del(8)(p21) and 6 MM patients with del(8)(p21) is displayed. Percentage of untreated live MM cells is considered as 100%. Statistical analysis of bortezomib and/or TRAIL treatment is assessed with Wilcoxon rank test in patients with or without del(8)(p21). * P<0.05, ** P<0.01, *** P<0.001. Mann-Whitney test was performed to compare patients groups with and without del(8)(p21). # P<0.05. Median values for each group are displayed in B and C.

**Fig 5:**
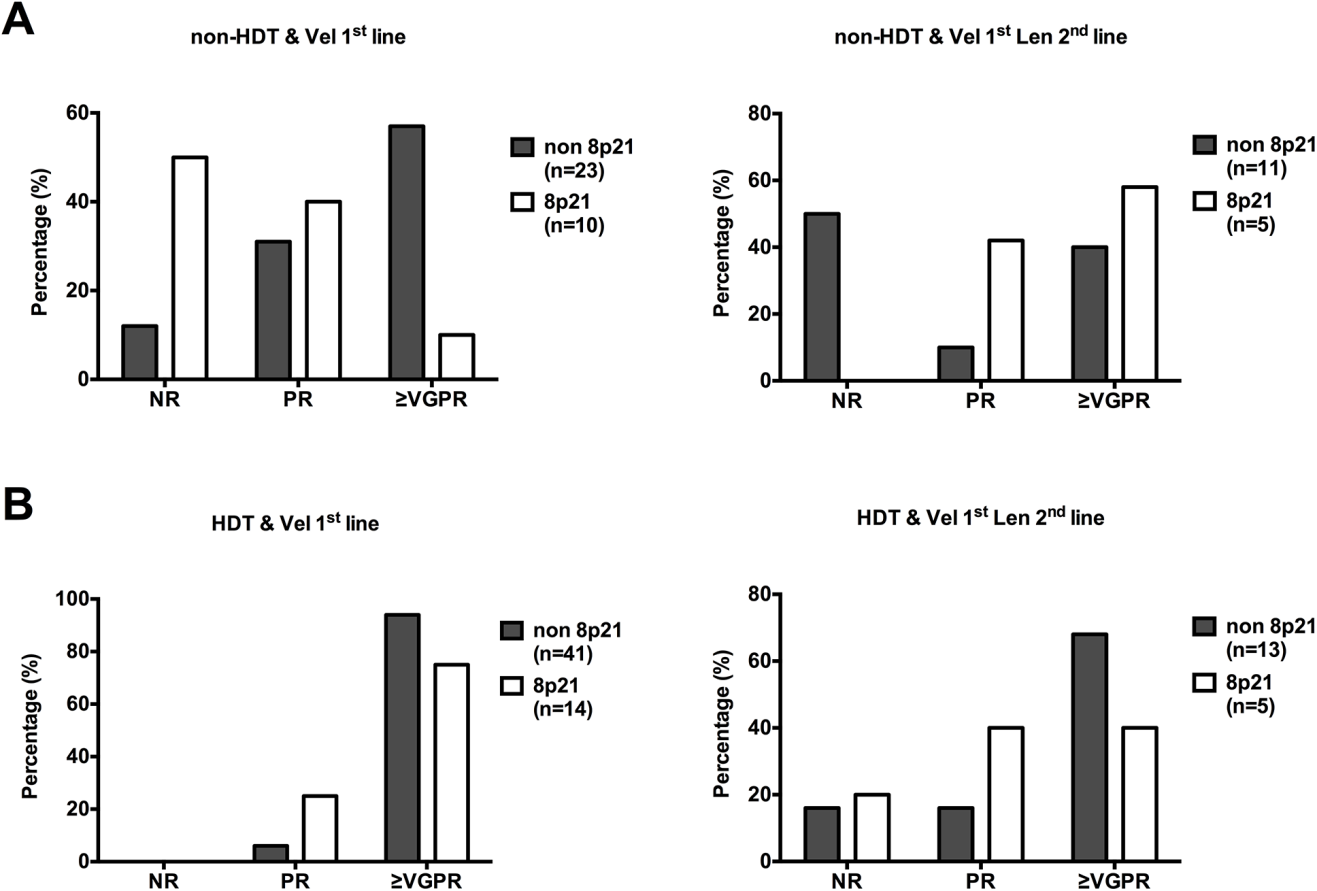
Patients with 8p21 deletion respond poorly to bortezomib treatment. Patients diagnosed with MM and receiving bortezomib based treatment at 1^st^ line were selected to this study (n = 88). (**A)** left panel is the non-HDT patients that received bortezomib (Vel: Velcade®, n = 33) as 1^st^ line treatment and right panel is the non-HDT patients that did not respond to 1^st^ line bortezomib treatment or relapsed and treated with Lenalidomide based regimen as a 2^nd^ line treatment (n = 16). B. Left panel displays patients received high dose treatment and bortezomib as 1^st^ line treatment (n = 55) and right panel is the response to Lenalidomide based treatment as 2^nd^ line for relapse patients (n = 18). Details of the treatments are shown in materials & methods section. Chi-square test was performed to analyze results.

Graphs and statistical analysis were done by GraphPad Prism (GraphPad Software Inc. La Jolla, CA, USA). Log-rank (Mantel-cox) test was used for Fig 1. Unpaired t-test was used to analyze Fig 2. Wilcoxon rank tests and Mann-Whitney tests were used to analyze the results for Figs 3 and 4. The data for Figs 3 and 4 are presented in S5 Table. Chi-square test was performed to analyze results in Fig 5.

## Results

### Deletion at the chromosomal region 8p21 results in poor survival

We have previously reported that the loss of 8p21region was significantly associated with poor progression-free survival (PFS) and overall survival (OS) in a cohort of MM patients that were diagnosed in our clinic [6]. Here, we analyze a larger cohort of patients that were diagnosed with multiple myeloma between 2008 and 2012 (37 with del(8)(p21) and 103 without deletion) (Table 1). Univariate analysis of patient data did not show any significant difference in any parameter at diagnosis except skeletal destruction. Patients with del(8)(p21) had significantly higher bone lesions (76%) compared to 55% in patients without the deletion (*p* = 0.027) (Table 1).

**Table 1:** Univariate analysis of clinical parameters of multiple myeloma patients.

	Patients with del(8)(p21)		Patients without del(8)(p21)		
	n	Mean (SD)	n	Mean (SD)	p value
Gender (male)	37	57%	103	39%	0.069
Age (y)	37	63 (10.3)	103	60 (10.8)	0.160
Bone disease (present)	37	76%	102	55%	**0.027**
s-Calcium	36	2.47 (0.31)	94	3.49 (10.2)	0.548
Hemoglobin	36	106 (23.1)	95	112 (21.0)	0.134
β_2_M	35	4.7 (4.07)	95	5.7 (11.4)	0.630
s-Albumin	36	34 (5.81)	94	32 (5.21)	0.121
s-Creatinine	36	141 (208)	99	123 (128)	0.542

In this cohort, 88 patients were treated with bortezomib as first line agent and we find that MM patients carrying 8p21 deletion had significantly shorter time to progression (TTP) compared to patients without the deletion (*p* = 0.0159) (Fig 1A) and most importantly these patients had significantly shorter overall survival (OS) compared to patients without the deletion (p = 0.0219) (Fig 1D), with a hazard ratio of 2.043 (1.18–4.74) and 2.96 (1.22–11.64), respectively. Furthermore, OS of myeloma patients carrying 8p21 deletion that underwent HDT had significantly decreased compared to patients without the deletion (Fig 1B and 1E). On the other hand, patients that are not eligible for HDT showed no significant difference in OS or TTP (Fig 1C and 1F).

In multivariate analysis del(8)(p21) presented as an independent prognostic factor associated with poor overall survival in agreement with our prior observations (Table 2). Consequently, these results confirm ours and others’ previous observations that del(8)(p21) negatively affects the prognosis of MM patients.

**Table 2:** Multivariate analysis of prognostic factors in multiple myeloma patients.

	OS		TTP	
	p	Relative risk (95% CI)	p	Relative risk (95% CI)
del(8)(p21)	**0.010**	4.904 (1.467–16.396)	0.187	1.635 (0.788–3.393)
gain(1q), t(4;14), t(14;16), del17p	0.270	1.969 (0.591–6.568)	0.600	1.187 (0.625–2.254)
s-Calcium	0.319	0.915 (0.769–1.089)	**0.005**	0.882 (0.809–0.962)
Hemoglobin	0.301	1.012 (0.989–1.036)	0.972	1.000 (0.986–1.015)
β2M	0.457	1.054 (0.918–1.209)	**0.004**	1.137 (1.042–1.241)
s-Albumin	0.135	0.920 (0.826–1.026)	0.906	0.996 (0.936–1.061)
s-Creatinine	0.736	0.999 (0.996–1.003)	0.233	0.998 (0.996–1.001)

### Deletion at the chromosomal region 8p21 leads to altered gene expression in primary MM cells

In order to identify the changes associated with del(8)(p21), expression levels of a certain set of genes were comparatively analyzed in patients with or without the deletion by quantitative real-time PCR (S1 Table).

When the two sets of patients were compared for the mRNA levels of these genes, several cases of differential expression were observed (Fig 2). At the mRNA level, patients with del(8)(p21) showed similar levels of expression for TRAIL-R1,-R2 and R3 while they showed significantly upregulated expression of TRAIL-R4 when compared to patients without del(8)(p21) (Fig 2A). These results indicate that while del(8)(p21) is expected to affect the expression levels of genes located in the region, the TRAIL receptor gene cluster is minimally affected, with no changes in mRNA levels of three genes and upregulation of the decoy receptor, TRAIL-R4.

As expected, the expression of several genes located on or near the 8p21 region were significantly altered in patients carrying del(8)(p21) (Fig 2B). Of the analyzed genes, DOCK5, IKBKB, CCDC25, CEBPD, RHOBTB2, KIAA1967, PHYHIP and SCARA3 showed statistically significant downregulation of at least two-fold. More interestingly, three of the analyzed genes located on 8p21 (TRAIL-R4, PTK2B and SLC39A14) showed at least two-fold upregulated mRNA levels despite the possible deletion of one gene copy.

Genes located elsewhere on the genome also showed differential expression in patients with del(8)(p21) compared to patients without the deletion. As presented in Fig 2C, genes with oncogenic potential such as MYC, BCL2 and RINT1 were upregulated while the tumor suppressor TP53 was downregulated. Genes involved in chromatin remodeling such as SMARCA4 (BRG1) and CHD3 as well as genes involved in immune function such as NFKB1 and LPXN also showed differential expression in patients with del(8)(p21).

In order to identify the effect of other commonly seen chromosomal aberrations, we analyzed the gene expression data with regards to other commonly observed chromosomal abnormalities in this cohort of patients (S2 Table). This analysis has revealed that the deregulated expression of the MYC oncogene is also associated with 1q21, 11q13, 15q22, 19q13 and 13q aberrations, the downregulation of KIAA1967 is also associated with chromosome 9q21, 11q13 and 15q22 aberrations and upregulation of BCL2 is also associated with 15q22 amplification.

### Patients with del(8)(p21) fail to upregulate pro-apoptotic TRAIL receptor expression upon bortezomib treatment

We next determined changes in the cell surface expression levels of the pro-apoptotic receptors TRAIL-R1 and R2, as well as the anti-apoptotic decoy receptors TRAIL-R3 and R4 upon *in vitro* bortezomib treatment in MM cells with or without del(8)(p21) (Fig 3).

CD138^+^ myeloma cells expressed similar levels of TRAIL-R1 regardless of del(8)(p21) status, in line with mRNA expression results. While a similar trend of TRAIL-R4 upregulation was observed in patients with del(8)(p21), this did not reach statistical significance possibly due to the use of a smaller sample size for flow cytometric phenotyping.

More interestingly, while 24 hour bortezomib treatment (10nM) could successfully upregulate TRAIL-R1 expression in MM cells of patients without del(8)(p21) (1.31 fold, p = 0.0059), MM cells from patients with del(8)(p21) failed to upregulate the pro-apoptotic TRAIL-R1 expression (1.11 fold, p = 0.1254) (Fig 3A). Furthermore, bortezomib treatment did not have any impact on expression levels of TRAIL-R2 in patients with (1.11 fold, p = 0.6739) or without (1.06 fold, p = 0.2165) del(8)(p21) (Fig 3B). Although bortezomib treatment of myeloma cells displayed a statistically non-significant trend of TRAIL-R3 upregulation in patients without 8p21 deletion (1.46 fold, p = 0.0697), it did not have any impact on cells carrying the deletion (0.92 fold, p = 0.1855)(Fig 3C). Likewise, the expression of TRAIL-R4 was not affected by bortezomib treatment regardless of del(8)(p21) status (no del(8)(p21) 1.14 fold, p = 0.2947; del(8)(p21) 0.81 fold, p = 0.2242) (Fig 3D).

Taken together, these data indicate that upon bortezomib exposure, the most important and significant difference is, unlike the cells without the deletion, MM cells carrying del(8)(p21) fail to upregulate the pro-apoptotic TRAIL-R1.

Changes observed in TRAIL receptor levels were mainly myeloma specific since expression of TRAIL receptors in CD138^-^/CD45^+^compartment of bone marrow did not get affected by bortezomib treatment irrespective of del(8)(p21) status (S1 Fig). Thus, these results indicate that bortezomib treatment could not efficiently sensitize myeloma cells with del(8)(p21) to TRAIL mediated apoptosis.

### Patients with del(8)(p21) are less sensitive to bortezomib and TRAIL mediated apoptosis

It has been previously demonstrated that bortezomib treatment can upregulate TRAIL receptors in various tumor types, which can result in sensitization of these cells to TRAIL mediated apoptosis[4, 15]. Here, sensitivity of myeloma cells with or without del(8)(p21) to bortezomib and soluble TRAIL/APO2L is assessed in order to evaluate the impact of differential gene expression driven by 8p21 deletion, such as TRAIL-R4, on myeloma cell survival. Downregulation of CD138, which is one of the early markers of apoptosis in myeloma cells and the percentage of CD38^+^/CD138^Bright^ primary MM cells[16] as well as the amount of viable, apoptotic and dead cells were assessed simultaneously by flow cytometry (Fig 4).

In myeloma cells without del(8)(p21), bortezomib alone and soluble TRAIL/APO2L alone efficiently induced a decrease in CD38^+^/CD138^Bright^ MM cells as well as apoptosis. More importantly, the combination of bortezomib and TRAIL treatment significantly increased apoptosis and decrease the number of CD38^+^/CD138^Bright^ MM cells (Fig 4B and 4C), implying that bortezomib treatment can further sensitize myeloma cells without 8p21 deletion to TRAIL-mediated killing.

While bortezomib treatment slightly affected the ratio of CD38^+^/CD138^Bright^ MM cells, it failed to induce apoptosis on myeloma cells with 8p21 deletion, either alone or in combination with soluble TRAIL/APO2L (Fig 4B and 4C). Additionally, we have employed a similar approach and analyzed the data by grouping the patients according to other commonly observed chromosomal abnormalities including amp11q13, amp1q21, amp15q22 and del13q. Only when the grouping was done according to 8p21 status, there was significant difference in the response to *in vitro* bortezomib and TRAIL (S2 Fig).

Thus, our *in vitro* analysis revealed that a higher proportion of cells from myeloma patients carrying the 8p21 deletion are less sensitive to both bortezomib and TRAIL mediated killing than myeloma cells from patients without the deletion. Therefore it is essential to assess the clinical impact of bortezomib treatment on MM patients with del(8)(p21).

### Patients with del(8)(p21) respond poorly to bortezomib treatment

During our *in vitro* observations that MM cells carrying the del(8)(p21) responded poorly to bortezomib or bortezomib mediated sensitization to TRAIL/APO2L killing, we sought to investigate whether this phenomenon showed any effect in the clinical response of patients carrying the deletion. In order to ascertain the effect of bortezomib alone, we have primarily investigated the response of patients that were not eligible for HDT and received bortezomib as a first-line treatment (Fig 5A). The clinical response to bortezomib as a first-line agent clearly demonstrates that patients with del(8)(p21) respond very poorly, with about half the patients showing no response (NR) at all and less than 10% of them showing very good partial remission (VGPR) or better. On the contrary, the patients without the deletion have a much better response to bortezomib, with more than 50% of the patients achieving VGPR or better and only about 10% of the patients showing NR (p = 0.0216). Interestingly, when the patients that are not eligible for HDT relapsed and received Lenalidomide as a second line treatment (Fig 5A), the response of patients with the deletion significantly surpassed those without the deletion (p = 0.0302), indicating that despite their poor response to bortezomib, patients with the deletion might still benefit greatly from Lenalidomide treatment if HDT is not an option. On the other hand, we have found that in patients receiving HDT, the negative effect of 8p21 deletion on patient response is largely, if not fully, overcome with a non significant difference in response between patients with and without del(8)(p21) (p = 0.2579) (Fig 5B). When patients receiving HDT relapsed and received Lenalidomide as a second-line treatment, there was little difference between the patient groups, with slightly lower levels of del(8)(p21) carrying patients achieving VGPR or better (p = 0.4640).

Taken together, these data indicate that patients with del(8)(p21) clinically have a poor response to bortezomib treatment, which might be possible to improve by using HDT or administration of immunomodulatory drugs such as Lenalidomide at an earlier stage of the disease.

## Discussion

Recent years have witnessed a significant increase in the survival rates for MM patients due to the introduction of combination therapies including proteasome inhibitors such as bortezomib and immunomodulatory drugs such as thalidomide, and lenalidomide [17]. Bortezomib has been proven to be the most efficient 1^st^ line therapy regimen but approximately 20% of patients still do not respond to bortezomib treatment and underlying mechanisms that lead to bortezomib resistance are currently being scrutinized in detail[18–20]. However, immunological aspects of this resistance have been largely omitted.

In this study, we have investigated the molecular consequences of 8p21 deletion, which is seen in approximately 20% of MM patients[5–8] and its potential link to resistance against bortezomib treatment. Since the exact position of the chromosomal break is unknown and possibly differs from patient to patient, we have selected for analysis, a set of genes located on or nearby the 8p21 region that might be essential for tumor survival and bortezomib resistance. This set of genes was further enlarged by the addition of other targets which showed differential expression in prior gene expression profiling studies on MM cells[13] and could be functionally linked to one or more genes located on or near the 8p21 region.

The pro-apoptotic TRAIL receptors, TRAIL-R1 and TRAIL-R2, as well as the decoy receptors TRAIL-R3 and TRAIL-R4 are located in the 8p21 region. Despite the expectation that losing one copy of the region would significantly diminish the expression of genes located here, we surprisingly did not observe any significant changes for TRAIL-R1, R2 and R3 at the mRNA level compared to patients without the deletion while an elevated expression of anti-apoptotic TRAIL-R4 was seen. We have observed the decreased expression of TP53[21] which directly affects TRAIL-R2 levels in myeloma cell lines[22] but decreased TP53 expression in MM cells carrying del(8)(p21) did not lead to downregulation of TRAIL-R2 expression neither at the mRNA level nor at the cell surface. Previous studies did not always indicate a direct correlation between TRAIL receptor expression and TRAIL mediated apoptosis in tumor models[15, 23, 24] since other factors such as the expression levels and activities of NFkB and BCL-2 also play important roles in bortezomib resistance[25–27] as well as TRAIL mediated apoptosis[12, 28, 29]. Here we show that both BCL-2 and NFkB mRNA levels are increased in MM cells with del(8)(p21) (Fig 2). Along with the high expression of the MYC oncogene that has been shown to promote MM cell survival[30–32], in MM cells carrying the 8p21 deletion, we have observed the upregulated expression of PTK2B *(Pyk2 or RAFTK)* which was recently demonstrated to promote tumor progression in MM[9]. Additionally, the diminished expression of SCARA3, which is negatively correlated with multiple myeloma prognosis[10] together with the aforementioned changes of gene expression in cells carrying the deletion might provide a platform that promotes myeloma cell survival.

It must be noted that, del(8)(p21) is commonly seen together with a range of other recurrent chromosomal abnormalities that might influence global gene expression profile. For 17p13 aberrations in the present cohort, we observed 2/19 of patients with del(8)(p21) carry a deletion and 1/6 of patients without del(8)(p21) carry an amplification while t(4;14) was observed in 2/19 of patients with del(8)(p21) and 1/6 of patients without del(8)(p21). Due to the low number of patients carrying these additional chromosomal abnormalities, the data presented in this study can neither confirm nor reject the presence of any effect that might be due to the global gene expression changes caused by del(17)(p13) or t(4;14)[33, 34].

Regarding other chromosomal abnormalities commonly seen in this cohort of patients, our analysis (S2 Table) reveals that the gene expression changes of MYC, KIAA1967 and BCL2 are also associated with the presence of other chromosomal abnormalities and cannot be attributed to del(8)(p21) alone.

In line with previous observations that bortezomib upregulates the expression of death receptors TRAIL-R1 and-R2[35–37], we observed that bortezomib treatment significantly upregulated cell surface expression of TRAIL-R1 in MM cells that do not carry the del(8)(p21), making them vulnerable to TRAIL mediated apoptosis. However, MM cells carrying del(8)(p21) failed to upregulate death receptors upon bortezomib treatment (Fig 3A and 3B) while keeping steady levels of pro-survival receptors TRAIL-R3 and-R4. Consequently, soluble TRAIL/APO2L and bortezomib failed to induce apoptosis efficiently whether used alone or in combination on MM cells carrying the deletion (Fig 4). It is essential to investigate the *in vitro* cytotoxic impact of other drugs, which are used in treatment of MM such as Melphalan and Dexamethasone, on myeloma cells carrying 8p21 deletion. It is noteworthy that in cells with del(8)(p21), a slight decrease in CD38^+^/CD138^Bright^ MM cells, which could be an early marker of apoptosis in myeloma cells, was observed. These results support the hypothesis that del(8)(p21) triggers an apoptotic resistance mechanism that uncouples CD138 downregulation from apoptotic cell death, so despite the decrease in the amount of CD38^+^/CD138^Bright^ MM cells, efficient triggering of apoptotic pathways is lacking.

It should also be noted that even though bortezomib has potential to sensitize tumor cells to TRAIL mediated apoptosis, it has also been shown to negatively affect T cell and NK cell function[38, 39]. Conversely, the immunomodulatory drug Lenalidomide upregulates TRAIL expression on NK cells, which might enhance TRAIL mediated killing of myeloma cells[40, 41]. Our clinical data also shows that while bortezomib largely fails to elicit a good response in patients carrying del(8)(p21), Lenalidomide treatment as second line reverts this negative effect (Fig 5).

To conclude, our results demonstrate that the changes associated with del(8)(p21) in MM, might be the foundation of resistance to bortezomib treatment and resistance against bortezomib mediated sensitization to TRAIL/APO2L killing. This is strongly supported by clinical data which demonstrates the poor response of the patients with del(8)(p21) to bortezomib as a first line treatment and calls for the incorporation of agents such as Lenalidomide into the treatment regimen in order to avert the negative impact of 8p21 deletion on patient survival, but should be further investigated with randomized trials.

## Supporting Information

S1 Fig Bortezomib does not alter TRAIL receptor expression of CD138^-^ BM cells. (DOCX)

S2 Fig Analysis of sensitivity to bortezomib and TRAIL mediated apoptosis within different chromosomal abnormalities. (DOCX)

S1 Table List of genes analyzed by quantitative RT-PCR. (DOCX)

S2 Table Analysis of gene expression data according to chromosomal abnormalities commonly seen in this patient cohort. (DOCX)

S3 Table Chromosomal abnormalities of patients used in TLDA mRNA analysis (DOCX)

S4 Table Chromosomal abnormalities of patient cells used in TRAIL-R cell surface expression and apoptosis assays. (DOCX)

S5 Table Summary of data for Figs 3 and 4. (DOCX)
