# Supplementary material for: Deletion of Chromosomal Region 8p21 Confers Resistance to Bortezomib and Is Associated with Upregulated Decoy TRAIL Receptor Expression in Patients with Multiple Myeloma
Source: PLoS One. 2015 Sep 17;10(9):e0138248. doi: 10.1371/journal.pone.0138248 (PMC4574561; doi:10.1371/journal.pone.0138248)
Supplement: S1 Fig — (DOCX) [file pone.0138248.s001.docx]

**S1 Figure**

**
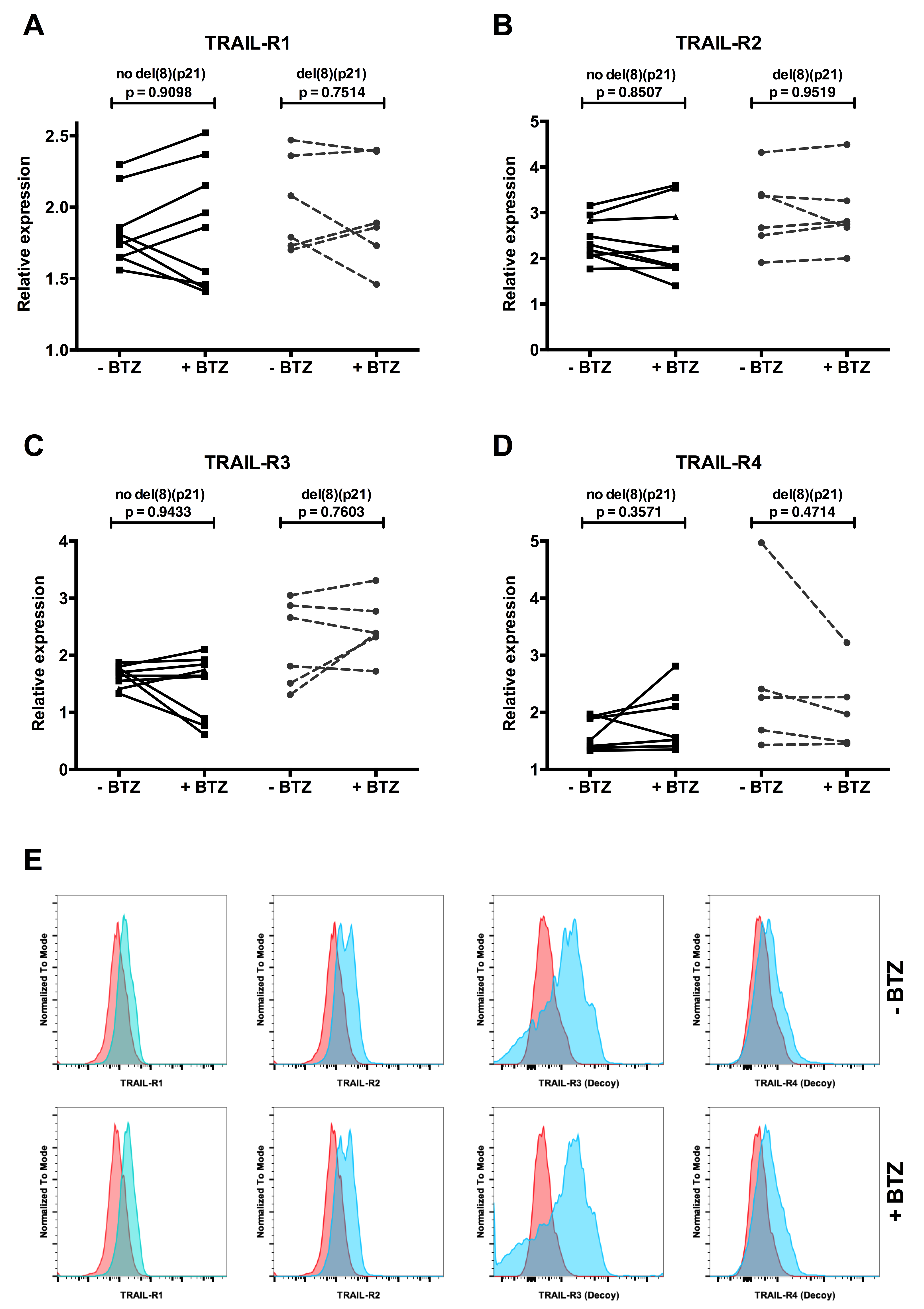
**

**TRAIL receptor expression in non-myeloma bone marrow cells:**

Relative expression of TRAIL receptors in non-myeloma cells (CD138^-^CD45^+^) of 6 MM patients with del(8)(p21) and 9 MM patients without deletion is determined by flow cytometry. Cell surface Expression levels are normalized to corresponding isotype controls. **(A)** TRAIL-R1, **(B)** TRAIL-R2, **(C)** TRAIL-R3, **(D)** TRAIL-R4. Effect of bortezomib treatment on TRAIL receptor expression is analyzed by paired t-test. **(E)** Representative histograms showing cell surface TRAIL receptor expression with or without Bortezomib. Red: isotype controls, blue: anti-TRAILR staining.
