# Supplementary material for: Deletion of Chromosomal Region 8p21 Confers Resistance to Bortezomib and Is Associated with Upregulated Decoy TRAIL Receptor Expression in Patients with Multiple Myeloma
Source: PLoS One. 2015 Sep 17;10(9):e0138248. doi: 10.1371/journal.pone.0138248 (PMC4574561; doi:10.1371/journal.pone.0138248)
Supplement: S2 Fig — (DOCX) [file pone.0138248.s002.docx]

**S2 Figure**

**
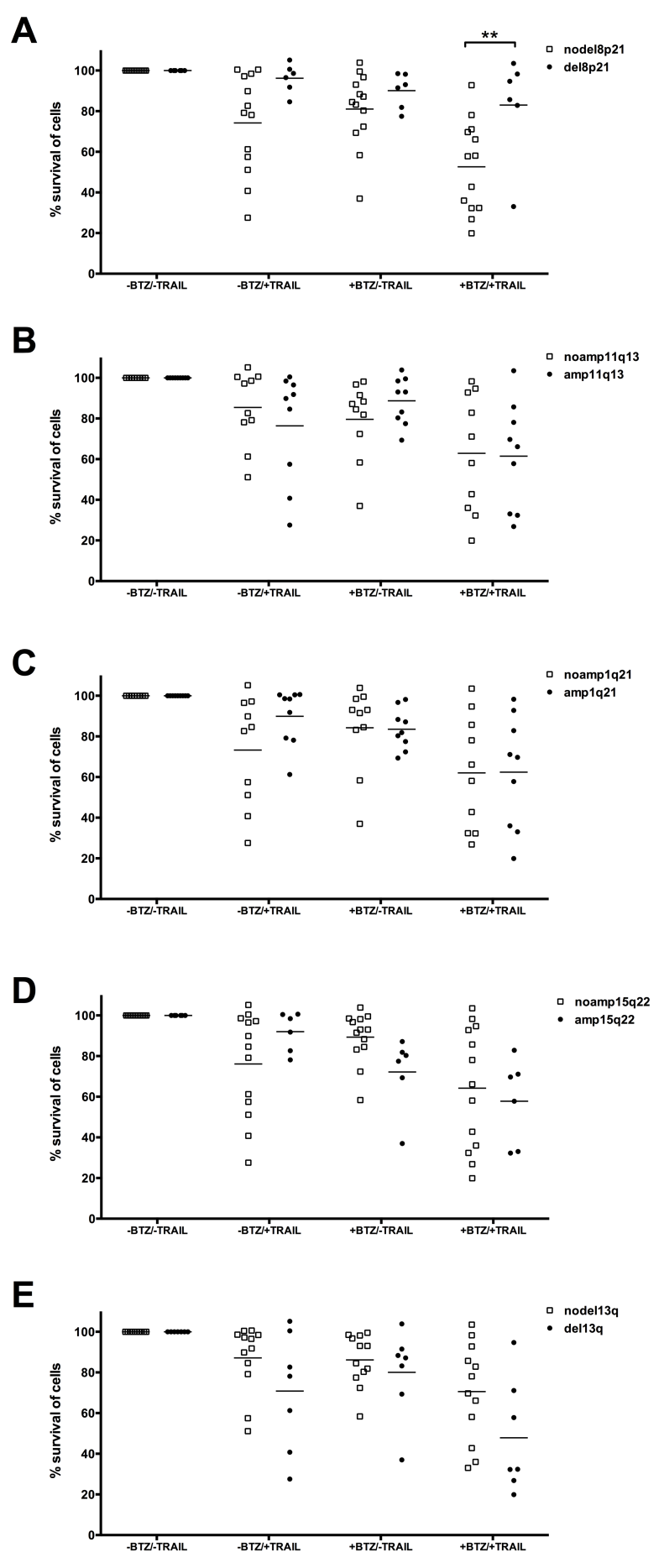
Patients with del(8)(p21) are less sensitive to bortezomib and TRAIL mediated apoptosis:** CD138 expression levels and Annexin V/PI staining was performed to assess viable, apoptotic and dead cells. Relative percentage of live MM cells (Annexin V^-^ and PI^-^ cells) of 19 MM patients is grouped according to **(A)** del(8)(p21), **(B)** amp(11)(q13), **(C)** amp(1q)(21), **(D)** amp(15)(q22) and (E) del(13)(q). Percentage of untreated live MM cells is considered as 100%. Statistical analysis of bortezomib and/or TRAIL treatment is assessed with paired t-test (** P<0.01).
