# Supplementary material for: Deletion of Chromosomal Region 8p21 Confers Resistance to Bortezomib and Is Associated with Upregulated Decoy TRAIL Receptor Expression in Patients with Multiple Myeloma
Source: PLoS One. 2015 Sep 17;10(9):e0138248. doi: 10.1371/journal.pone.0138248 (PMC4574561; doi:10.1371/journal.pone.0138248)
Supplement: S1 Table — (DOCX) [file pone.0138248.s003.docx]

**S1 Table. List of genes analyzed by quantitative RT-PCR.**

| **Genes that were significantly upregulated in MM cells with del(8)(p21) with respect to MM cells without del(8)(p21) (p<0.05, unpaired t-test)** | | | | | |  | |  |  |
| --- | --- | --- | --- | --- | --- | --- | --- | --- | --- |
| **Gene ID** | **Assay ID** | **Gene Symbol** | **Name** | **Chromosomal Region** | **Fold change** |  |  |  |  |
| [4609](http://www.ncbi.nlm.nih.gov/sites/entrez?cmd=retrieve&db=gene&list_uids=4609&dopt=full_report) | Hs00153408_m1 | MYC | v-myc myelocytomatosis viral oncogene homolog (avian) | **8q24.21** | **6,692** |  |  |  |  |
| [596](http://www.ncbi.nlm.nih.gov/sites/entrez?cmd=retrieve&db=gene&list_uids=596&dopt=full_report) | Hs00153350_m1 | BCL2 | B-cell CLL/lymphoma 2 | **18q21.3** | **6,219** |  |  |  |  |
| [8793](http://www.ncbi.nlm.nih.gov/sites/entrez?cmd=retrieve&db=gene&list_uids=8793&dopt=full_report) | Hs00174664_m1 | TRAIL-R4 | tumor necrosis factor receptor superfamily, member 10d, decoy with truncated death domain | **8p21** | **5,764** |  |  |  |  |
| [23516](http://www.ncbi.nlm.nih.gov/sites/entrez?cmd=retrieve&db=gene&list_uids=23516&dopt=full_report) | Hs00299262_m1 | SLC39A14 | solute carrier family 39 (zinc transporter), member 14 | **8p21.3** | **3,399** |  |  |  |  |
| [2185](http://www.ncbi.nlm.nih.gov/sites/entrez?cmd=retrieve&db=gene&list_uids=2185&dopt=full_report) | Hs00169444_m1 | PTK2B | PTK2B protein tyrosine kinase 2 beta | **8p21.1** | **3,317** |  |  |  |  |
| [4790](http://www.ncbi.nlm.nih.gov/sites/entrez?cmd=retrieve&db=gene&list_uids=4790&dopt=full_report) | Hs00231653_m1 | NFKB1 | nuclear factor of kappa light polypeptide gene enhancer in B-cells 1 | **4q24** | **3,017** |  |  |  |  |
| [6597](http://www.ncbi.nlm.nih.gov/sites/entrez?cmd=retrieve&db=gene&list_uids=6597&dopt=full_report) | Hs00231324_m1 | SMARCA4 | SWI/SNF related, matrix associated, actin dependent regulator of chromatin, subfamily a, member 4 | **19p13.2** | **2,809** |  |  |  |  |
| [60561](http://www.ncbi.nlm.nih.gov/sites/entrez?cmd=retrieve&db=gene&list_uids=60561&dopt=full_report) | Hs00222515_m1 | RINT1 | RAD50 interactor 1 | **7q22.2** | **2,682** |  |  |  |  |
| [9404](http://www.ncbi.nlm.nih.gov/sites/entrez?cmd=retrieve&db=gene&list_uids=9404&dopt=full_report) | Hs00183105_m1 | LPXN | leupaxin | **11q12.1** | **2,353** |  |  |  |  |
|  |  |  |  |  |  |  |  |  |  |
| **Genes that were significantly downregulated in MM cells with del(8)(p21) with respect to MM cells without del(8)(p21) (p<0.05, unpaired t-test)** | | | | | |  | |  |  |
| **Gene ID** | **Assay ID** | **Gene Symbol** | **Name** | **Chromosomal Region** | **Fold change** |  |  |  |  |
| [9796](http://www.ncbi.nlm.nih.gov/sites/entrez?cmd=retrieve&db=gene&list_uids=9796&dopt=full_report) | Hs00901974_m1 | PHYHIP | phytanoyl-CoA 2-hydroxylase interacting protein | **8p21.3** | **0,034** |  |  |  |  |
| [51435](http://www.ncbi.nlm.nih.gov/sites/entrez?cmd=retrieve&db=gene&list_uids=51435&dopt=full_report) | Hs00939871_m1 | SCARA3 | scavenger receptor class A, member 3 | **8p21** | **0,051** |  |  |  |  |
| [57805](http://www.ncbi.nlm.nih.gov/sites/entrez?cmd=retrieve&db=gene&list_uids=57805&dopt=full_report) | Hs00368356_m1 | KIAA1967 | KIAA1967 | **8p22** | **0,067** |  |  |  |  |
| [23221](http://www.ncbi.nlm.nih.gov/sites/entrez?cmd=retrieve&db=gene&list_uids=23221&dopt=full_report) | Hs01598095_g1 | RHOBTB2 | Rho-related BTB domain containing 2 | **8p21.3** | **0,091** |  |  |  |  |
| [1052](http://www.ncbi.nlm.nih.gov/sites/entrez?cmd=retrieve&db=gene&list_uids=1052&dopt=full_report) | Hs00270931_s1 | CEBPD | CCAAT/enhancer binding protein (C/EBP), delta | **8p11.2-p11.1** | **0,148** |  |  |  |  |
| [55246](http://www.ncbi.nlm.nih.gov/sites/entrez?cmd=retrieve&db=gene&list_uids=55246&dopt=full_report) | Hs00560350_m1 | CCDC25 | coiled-coil domain containing 25 | **8p21.1** | **0,243** |  |  |  |  |
| [3551](http://www.ncbi.nlm.nih.gov/sites/entrez?cmd=retrieve&db=gene&list_uids=3551&dopt=full_report) | Hs01559464_g1 | IKBKB | inhibitor of kappa light polypeptide gene enhancer in B-cells, kinase beta | **8p11.2** | **0,285** |  |  |  |  |
| [1107](http://www.ncbi.nlm.nih.gov/sites/entrez?cmd=retrieve&db=gene&list_uids=1107&dopt=full_report) | Hs01050221_g1 | CHD3 | chromodomain helicase DNA binding protein 3 | **17p13.1** | **0,381** |  |  |  |  |
| [80005](http://www.ncbi.nlm.nih.gov/sites/entrez?cmd=retrieve&db=gene&list_uids=80005&dopt=full_report) | Hs00227848_m1 | DOCK5 | dedicator of cytokinesis 5 | **8p21.1** | **0,413** |  |  |  |  |
| [7157](http://www.ncbi.nlm.nih.gov/sites/entrez?cmd=retrieve&db=gene&list_uids=7157&dopt=full_report) | Hs01034254_g1 | TP53 | tumor protein p53 | **17p13** | **0,540** |  |  |  |  |
|  |  |  |  |  |  |  |  |  |  |
| **Genes that did not show significant changes in mRNA levels (p>0.05, unpaired t-test)** | | | | | | |  | |  |
| **Gene ID** | **Assay ID** | **Gene Symbol** | **Name** | **Chromosomal Region** |  |  |  |  |  |
| [2597](http://www.ncbi.nlm.nih.gov/sites/entrez?cmd=retrieve&db=gene&list_uids=2597&dopt=full_report) | Hs02758991_g1 | GAPDH | glyceraldehyde-3-phosphate dehydrogenase | **12p13** |  |  |  |  |  |
| [328](http://www.ncbi.nlm.nih.gov/sites/entrez?cmd=retrieve&db=gene&list_uids=328&dopt=full_report) | Hs00172396_m1 | APEX1 | APEX nuclease (multifunctional DNA repair enzyme) 1 | **14q11.2-q12** |  |  |  |  |  |
| [4088](http://www.ncbi.nlm.nih.gov/sites/entrez?cmd=retrieve&db=gene&list_uids=4088&dopt=full_report) | Hs00232219_m1 | SMAD3 | SMAD family member 3 | **15q22.33** |  |  |  |  |  |
| [51588](http://www.ncbi.nlm.nih.gov/sites/entrez?cmd=retrieve&db=gene&list_uids=51588&dopt=full_report) | Hs00185538_m1 | PIAS4 | protein inhibitor of activated STAT, 4 | **19p13.3** |  |  |  |  |  |
| [5518](http://www.ncbi.nlm.nih.gov/sites/entrez?cmd=retrieve&db=gene&list_uids=5518&dopt=full_report) | Hs00204426_m1 | PPP2R1A | protein phosphatase 2 (formerly 2A), regulatory subunit A, alpha isoform | **19q13.33** |  |  |  |  |  |
| [3805](http://www.ncbi.nlm.nih.gov/sites/entrez?cmd=retrieve&db=gene&list_uids=3805&dopt=full_report) | Hs00427106_m1 | KIR2DL4 | killer cell immunoglobulin-like receptor, two domains, long cytoplasmic tail, 4 | **19q13.4** |  |  |  |  |  |
| [5111](http://www.ncbi.nlm.nih.gov/sites/entrez?cmd=retrieve&db=gene&list_uids=5111&dopt=full_report) | Hs99999177_g1 | PCNA | proliferating cell nuclear antigen | **20pter-p12** |  |  |  |  |  |
| [598](http://www.ncbi.nlm.nih.gov/sites/entrez?cmd=retrieve&db=gene&list_uids=598&dopt=full_report) | Hs99999146_m1 | BCL2L1 | BCL2-like 1 | **20q11.21** |  |  |  |  |  |
| [25793](http://www.ncbi.nlm.nih.gov/sites/entrez?cmd=retrieve&db=gene&list_uids=25793&dopt=full_report) | Hs00201825_m1 | FBXO7 | F-box protein 7 | **22q12-q13** |  |  |  |  |  |
| [10015](http://www.ncbi.nlm.nih.gov/sites/entrez?cmd=retrieve&db=gene&list_uids=10015&dopt=full_report) | Hs00183813_m1 | PDCD6IP | programmed cell death 6 interacting protein | **3p22.3** |  |  |  |  |  |
| [7015](http://www.ncbi.nlm.nih.gov/sites/entrez?cmd=retrieve&db=gene&list_uids=7015&dopt=full_report) | Hs99999022_m1 | TERT | telomerase reverse transcriptase | **5p15.33** |  |  |  |  |  |
| [60](http://www.ncbi.nlm.nih.gov/sites/entrez?cmd=retrieve&db=gene&list_uids=60&dopt=full_report) | Hs03023943_g1 | ACTB | actin, beta | **7p15-p12** |  |  |  |  |  |
| [6867](http://www.ncbi.nlm.nih.gov/sites/entrez?cmd=retrieve&db=gene&list_uids=6867&dopt=full_report) | Hs00180691_m1 | TACC1 | transforming, acidic coiled-coil containing protein 1 | **8p11** |  |  |  |  |  |
| [286](http://www.ncbi.nlm.nih.gov/sites/entrez?cmd=retrieve&db=gene&list_uids=286&dopt=full_report) | Hs00252830_m1 | ANK1 | ankyrin 1, erythrocytic | **8p11.1** |  |  |  |  |  |
| [11212](http://www.ncbi.nlm.nih.gov/sites/entrez?cmd=retrieve&db=gene&list_uids=11212&dopt=full_report) | Hs00200497_m1 | PROSC | proline synthetase co-transcribed homolog (bacterial) | **8p11.2** |  |  |  |  |  |
| [2260](http://www.ncbi.nlm.nih.gov/sites/entrez?cmd=retrieve&db=gene&list_uids=2260&dopt=full_report) | Hs00241111_m1 | FGFR1 | fibroblast growth factor receptor 1 | **8p11.2-p11.1** |  |  |  |  |  |
| [9530](http://www.ncbi.nlm.nih.gov/sites/entrez?cmd=retrieve&db=gene&list_uids=9530&dopt=full_report) | Hs00362193_m1 | BAG4 | BCL2-associated athanogene 4 | **8p12** |  |  |  |  |  |
| [3084](http://www.ncbi.nlm.nih.gov/sites/entrez?cmd=retrieve&db=gene&list_uids=3084&dopt=full_report) | Hs00247620_m1 | NRG1 | neuregulin 1 | **8p12** |  |  |  |  |  |
| [6422](http://www.ncbi.nlm.nih.gov/sites/entrez?cmd=retrieve&db=gene&list_uids=6422&dopt=full_report) | Hs00610060_m1 | SFRP1 | secreted frizzled-related protein 1 | **8p12-11.1** |  |  |  |  |  |
| [7486](http://www.ncbi.nlm.nih.gov/sites/entrez?cmd=retrieve&db=gene&list_uids=7486&dopt=full_report) | Hs00172155_m1 | WRN | Werner syndrome | **8p12-p11.2** |  |  |  |  |  |
| [665](http://www.ncbi.nlm.nih.gov/sites/entrez?cmd=retrieve&db=gene&list_uids=665&dopt=full_report) | Hs00188949_m1 | BNIP3L | BCL2/adenovirus E1B 19kDa interacting protein 3-like | **8p21** |  |  |  |  |  |
| [1135](http://www.ncbi.nlm.nih.gov/sites/entrez?cmd=retrieve&db=gene&list_uids=1135&dopt=full_report) | Hs00181237_m1 | CHRNA2 | cholinergic receptor, nicotinic, alpha 2 (neuronal) | **8p21** |  |  |  |  |  |
| [4747](http://www.ncbi.nlm.nih.gov/sites/entrez?cmd=retrieve&db=gene&list_uids=4747&dopt=full_report) | Hs00196245_m1 | NEFL | neurofilament, light polypeptide | **8p21** |  |  |  |  |  |
| [4824](http://www.ncbi.nlm.nih.gov/sites/entrez?cmd=retrieve&db=gene&list_uids=4824&dopt=full_report) | Hs00171834_m1 | NKX3-1 | NK3 homeobox 1 | **8p21** |  |  |  |  |  |
| [5368](http://www.ncbi.nlm.nih.gov/sites/entrez?cmd=retrieve&db=gene&list_uids=5368&dopt=full_report) | Hs00173823_m1 | PNOC | prepronociceptin | **8p21** |  |  |  |  |  |
| [8797](http://www.ncbi.nlm.nih.gov/sites/entrez?cmd=retrieve&db=gene&list_uids=8797&dopt=full_report) | Hs00269491_m1 | TRAIL-R1 | tumor necrosis factor receptor superfamily, member 10a | **8p21** |  |  |  |  |  |
| [2039](http://www.ncbi.nlm.nih.gov/sites/entrez?cmd=retrieve&db=gene&list_uids=2039&dopt=full_report) | Hs00157387_m1 | EPB49 | erythrocyte membrane protein band 4.9 (dematin) | **8p21.1** |  |  |  |  |  |
| [10863](http://www.ncbi.nlm.nih.gov/sites/entrez?cmd=retrieve&db=gene&list_uids=10863&dopt=full_report) | Hs00248020_m1 | ADAM28 | ADAM metallopeptidase domain 28 | **8p21.2** |  |  |  |  |  |
| [64641](http://www.ncbi.nlm.nih.gov/sites/entrez?cmd=retrieve&db=gene&list_uids=64641&dopt=full_report) | Hs00224081_m1 | EBF2 | early B-cell factor 2 | **8p21.2** |  |  |  |  |  |
| [5520](http://www.ncbi.nlm.nih.gov/sites/entrez?cmd=retrieve&db=gene&list_uids=5520&dopt=full_report) | Hs00160392_m1 | PPP2R2A | protein phosphatase 2 (formerly 2A), regulatory subunit B, alpha isoform | **8p21.2** |  |  |  |  |  |
| [81551](http://www.ncbi.nlm.nih.gov/sites/entrez?cmd=retrieve&db=gene&list_uids=81551&dopt=full_report) | Hs00229288_m1 | STMN4 | stathmin-like 4 | **8p21.2** |  |  |  |  |  |
| [649](http://www.ncbi.nlm.nih.gov/sites/entrez?cmd=retrieve&db=gene&list_uids=649&dopt=full_report) | Hs00986789_m1 | BMP1 | bone morphogenetic protein 1 | **8p21.3** |  |  |  |  |  |
| [91782](http://www.ncbi.nlm.nih.gov/sites/entrez?cmd=retrieve&db=gene&list_uids=91782&dopt=full_report) | Hs00292844_m1 | CHMP7 | CHMP family, member 7 | **8p21.3** |  |  |  |  |  |
| [9046](http://www.ncbi.nlm.nih.gov/sites/entrez?cmd=retrieve&db=gene&list_uids=9046&dopt=full_report) | Hs00182758_m1 | DOK2 | docking protein 2, 56kDa | **8p21.3** |  |  |  |  |  |
| [203190](http://www.ncbi.nlm.nih.gov/sites/entrez?cmd=retrieve&db=gene&list_uids=203190&dopt=full_report) | Hs00373763_m1 | LGI3 | leucine-rich repeat LGI family, member 3 | **8p21.3** |  |  |  |  |  |
| [5533](http://www.ncbi.nlm.nih.gov/sites/entrez?cmd=retrieve&db=gene&list_uids=5533&dopt=full_report) | Hs00194467_m1 | PPP3CC | protein phosphatase 3 (formerly 2B), catalytic subunit, gamma isoform | **8p21.3** |  |  |  |  |  |
| [10395](http://www.ncbi.nlm.nih.gov/sites/entrez?cmd=retrieve&db=gene&list_uids=10395&dopt=full_report) | Hs00183436_m1 | DLC1 | deleted in liver cancer 1 | **8p22** |  |  |  |  |  |
| [11178](http://www.ncbi.nlm.nih.gov/sites/entrez?cmd=retrieve&db=gene&list_uids=11178&dopt=full_report) | Hs00232762_m1 | LZTS1 | leucine zipper, putative tumor suppressor 1 | **8p22** |  |  |  |  |  |
| [1808](http://www.ncbi.nlm.nih.gov/sites/entrez?cmd=retrieve&db=gene&list_uids=1808&dopt=full_report) | Hs00954558_m1 | DPYSL2 | dihydropyrimidinase-like 2 | **8p22-p21** |  |  |  |  |  |
| [8795](http://www.ncbi.nlm.nih.gov/sites/entrez?cmd=retrieve&db=gene&list_uids=8795&dopt=full_report) | Hs00366278_m1 | TRAIL-R2 | tumor necrosis factor receptor superfamily, member 10b | **8p22-p21** |  |  |  |  |  |
| [8794](http://www.ncbi.nlm.nih.gov/sites/entrez?cmd=retrieve&db=gene&list_uids=8794&dopt=full_report) | Hs00182570_m1 | TRAIL-R3 | tumor necrosis factor receptor superfamily, member 10c, decoy without an intracellular domain | **8p22-p21** |  |  |  |  |  |
| [1960](http://www.ncbi.nlm.nih.gov/sites/entrez?cmd=retrieve&db=gene&list_uids=1960&dopt=full_report) | Hs00231780_m1 | EGR3 | early growth response 3 | **8p23-p21** |  |  |  |  |  |
| [23362](http://www.ncbi.nlm.nih.gov/sites/entrez?cmd=retrieve&db=gene&list_uids=23362&dopt=full_report) | Hs00209633_m1 | PSD3 | pleckstrin and Sec7 domain containing 3 | **8pter-p23.3** |  |  |  |  |  |
