# Supplementary material for: Deletion of Chromosomal Region 8p21 Confers Resistance to Bortezomib and Is Associated with Upregulated Decoy TRAIL Receptor Expression in Patients with Multiple Myeloma
Source: PLoS One. 2015 Sep 17;10(9):e0138248. doi: 10.1371/journal.pone.0138248 (PMC4574561; doi:10.1371/journal.pone.0138248)
Supplement: S2 Table — (DOCX) [file pone.0138248.s004.docx]

**S2 Table. Analysis of gene expression data according to chromosomal abnormalities commonly seen in this patient cohort.**

|  |  |  |  | amp(1)(q21)  (n=10) | |  | amp(9)  (n=10) | |  | amp(11)(q13)  (n=11) | |  | amp(15)(q22)  (n=12) | |  | amp(19)(q13)  (n=10) | |  | del(13q)  (n=11) | | |
| --- | --- | --- | --- | --- | --- | --- | --- | --- | --- | --- | --- | --- | --- | --- | --- | --- | --- | --- | --- | --- | --- |
| **Gene ID** | **Gene name** | **Assay ID** | **Chromosomal Region** | **Fold** | **P-value** |  | **Fold** | **P-value** |  | **Fold** | **P-value** |  | **Fold** | **P-value** |  | **Fold** | **P-value** |  | **Fold** | **P-value** |  |
| [4609](http://www.ncbi.nlm.nih.gov/sites/entrez?cmd=retrieve&db=gene&list_uids=4609&dopt=full_report) | MYC | Hs00153408_m1 | **8q24.21** | **6.113** | **<0.001** |  | 1.697 | ns |  | **4.148** | **<0.001** |  | **6.222** | **<0.001** |  | **3.865** | **<0.001** |  | **0.176** | **<0.001** |  |
| [596](http://www.ncbi.nlm.nih.gov/sites/entrez?cmd=retrieve&db=gene&list_uids=596&dopt=full_report) | BCL2 | Hs00153350_m1 | **18q21.3** | 1.823 | ns |  | 1.676 | ns |  | 2.329 | ns |  | **2.844** | **<0.05** |  | 3.578 | ns |  | 0.403 | ns |  |
| [8793](http://www.ncbi.nlm.nih.gov/sites/entrez?cmd=retrieve&db=gene&list_uids=8793&dopt=full_report) | TRAIL-R4 | Hs00174664_m1 | **8p21** | 0.800 | ns |  | 2.110 | ns |  | 1.998 | ns |  | 2.274 | ns |  | 3.582 | ns |  | 0.852 | ns |  |
| [23516](http://www.ncbi.nlm.nih.gov/sites/entrez?cmd=retrieve&db=gene&list_uids=23516&dopt=full_report) | SLC39A14 | Hs00299262_m1 | **8p21.3** | 0.736 | ns |  | 1.565 | ns |  | 1.410 | ns |  | 1.655 | ns |  | 1.562 | ns |  | 0.919 | ns |  |
| [2185](http://www.ncbi.nlm.nih.gov/sites/entrez?cmd=retrieve&db=gene&list_uids=2185&dopt=full_report) | PTK2B | Hs00169444_m1 | **8p21.1** | 1.220 | ns |  | 0.596 | ns |  | 1.104 | ns |  | 0.969 | ns |  | 1.042 | ns |  | 1.531 | ns |  |
| [4790](http://www.ncbi.nlm.nih.gov/sites/entrez?cmd=retrieve&db=gene&list_uids=4790&dopt=full_report) | NFKB1 | Hs00231653_m1 | **4q24** | 1.055 | ns |  | 1.987 | ns |  | 1.883 | ns |  | 2.042 | ns |  | 2.274 | ns |  | 0.791 | ns |  |
| [6597](http://www.ncbi.nlm.nih.gov/sites/entrez?cmd=retrieve&db=gene&list_uids=6597&dopt=full_report) | SMARCA4 | Hs00231324_m1 | **19p13.2** | 1.617 | ns |  | 1.445 | ns |  | 1.829 | ns |  | 2.241 | ns |  | 1.571 | ns |  | 0.596 | ns |  |
| [60561](http://www.ncbi.nlm.nih.gov/sites/entrez?cmd=retrieve&db=gene&list_uids=60561&dopt=full_report) | RINT1 | Hs00222515_m1 | **7q22.2** | 1.006 | ns |  | 1.606 | ns |  | 1.864 | ns |  | 1.695 | ns |  | 1.540 | ns |  | 0.730 | ns |  |
| [9404](http://www.ncbi.nlm.nih.gov/sites/entrez?cmd=retrieve&db=gene&list_uids=9404&dopt=full_report) | LPXN | Hs00183105_m1 | **11q12.1** | 1.126 | ns |  | 1.295 | ns |  | 1.573 | ns |  | 1.483 | ns |  | 1.329 | ns |  | 0.511 | ns |  |
| [9796](http://www.ncbi.nlm.nih.gov/sites/entrez?cmd=retrieve&db=gene&list_uids=9796&dopt=full_report) | PHYHIP | Hs00901974_m1 | **8p21.3** | 0.077 | ns |  | 0.074 | ns |  | 0.094 | ns |  | 0.116 | ns |  | 4.876 | ns |  | 0.077 | ns |  |
| [51435](http://www.ncbi.nlm.nih.gov/sites/entrez?cmd=retrieve&db=gene&list_uids=51435&dopt=full_report) | SCARA3 | Hs00939871_m1 | **8p21** | 0.089 | ns |  | 0.095 | ns |  | 0.147 | ns |  | 0.138 | ns |  | 0.383 | ns |  | 0.089 | ns |  |
| [57805](http://www.ncbi.nlm.nih.gov/sites/entrez?cmd=retrieve&db=gene&list_uids=57805&dopt=full_report) | KIAA1967 | Hs00368356_m1 | **8p22** | 0.089 | ns |  | **0.087** | **<0.05** |  | **0.076** | **<0.01** |  | **0.088** | **<0.05** |  | 0.718 | ns |  | 0.089 | ns |  |
| [23221](http://www.ncbi.nlm.nih.gov/sites/entrez?cmd=retrieve&db=gene&list_uids=23221&dopt=full_report) | RHOBTB2 | Hs01598095_g1 | **8p21.3** | 0.195 | ns |  | 0.186 | ns |  | 0.144 | ns |  | 0.166 | ns |  | 0.806 | ns |  | 0.195 | ns |  |
| [1052](http://www.ncbi.nlm.nih.gov/sites/entrez?cmd=retrieve&db=gene&list_uids=1052&dopt=full_report) | CEBPD | Hs00270931_s1 | **8p11.2-p11.1** | 0.214 | ns |  | 0.223 | ns |  | 0.287 | ns |  | 0.255 | ns |  | 0.468 | ns |  | 0.214 | ns |  |
| [55246](http://www.ncbi.nlm.nih.gov/sites/entrez?cmd=retrieve&db=gene&list_uids=55246&dopt=full_report) | CCDC25 | Hs00560350_m1 | **8p21.1** | 0.306 | ns |  | 0.307 | ns |  | 0.275 | ns |  | 0.273 | ns |  | 0.933 | ns |  | 0.306 | ns |  |
| [3551](http://www.ncbi.nlm.nih.gov/sites/entrez?cmd=retrieve&db=gene&list_uids=3551&dopt=full_report) | IKBKB | Hs01559464_g1 | **8p11.2** | 0.475 | ns |  | 0.547 | ns |  | 0.523 | ns |  | 0.575 | ns |  | 0.559 | ns |  | 0.475 | ns |  |
| [1107](http://www.ncbi.nlm.nih.gov/sites/entrez?cmd=retrieve&db=gene&list_uids=1107&dopt=full_report) | CHD3 | Hs01050221_g1 | **17p13.1** | 0.793 | ns |  | 0.662 | ns |  | 0.708 | ns |  | 0.388 | ns |  | 1.045 | ns |  | 0.793 | ns |  |
| [80005](http://www.ncbi.nlm.nih.gov/sites/entrez?cmd=retrieve&db=gene&list_uids=80005&dopt=full_report) | DOCK5 | Hs00227848_m1 | **8p21.1** | 0.582 | ns |  | 0.474 | ns |  | 1.021 | ns |  | 1.153 | ns |  | 0.955 | ns |  | 0.582 | ns |  |
| [7157](http://www.ncbi.nlm.nih.gov/sites/entrez?cmd=retrieve&db=gene&list_uids=7157&dopt=full_report) | TP53 | Hs01034254_g1 | **17p13** | 0.710 | ns |  | 0.601 | ns |  | 0.624 | ns |  | 0.737 | ns |  | 0.856 | ns |  | 0.710 | ns |  |

For those chromosomal aberrations that were commonly seen within the present patient cohort, analysis of RT-PCR based gene expression data was carried our by grouping patients into those that carry the abnormality and those that do not. “n” is the number of patients carrying the given chromosomal abnormality in each column and fold expression is calculated relative to the patients that do not carry the abnormality. Genes that were significantly affected by del(8)(p21) as in Supplementary Table I are shown- None of the other analyzed genes showed any significant differential expression when grouped according to these chromosomal abnormalities. (amp: amplification, del: deletion, ns: not significant, two-way ANOVA test)
