# Supplementary material for: Deletion of Chromosomal Region 8p21 Confers Resistance to Bortezomib and Is Associated with Upregulated Decoy TRAIL Receptor Expression in Patients with Multiple Myeloma
Source: PLoS One. 2015 Sep 17;10(9):e0138248. doi: 10.1371/journal.pone.0138248 (PMC4574561; doi:10.1371/journal.pone.0138248)
Supplement: S3 Table — (DOCX) [file pone.0138248.s005.docx]

**S3 Table. Chromosomal abnormalities of patients used in TLDA mRNA analysis**

Detailed cytogenetic characterization of the patient cohort used in the study. “+” indicates one additional signal (amplification) of the analyzed chromosomal region while x2 indicates 2 additional signals and “-” indicates loss of one signal.

| Patient code | del(8)p(21) (%) | Other chromosomal abnormalities |
| --- | --- | --- |
| MM1 | 90% | +1q21, +9q21, +11q13, +15q22, +19q13 |
| MM2 | 94% | +9q21, +11q13, +15q22, -14q32 |
| MM3 | 89% | +4p16, -6q21, +11q13, +15q22, -16q23, -17p13 |
| MM4 | 78% | -13q, t(11;14) |
| MM5 | 97% | +1q21,-13q |
| MM6 | 91% | +9q21, +11q13x2, -13q |
| MM7 | 63% | +9q21, +11q13x2, -13q, +15q22x2 +19q13x2 |
| MM8 | 89% | +9q21, +11q13, -13q, +15q22x3, +19q13 |
| MM9 | 80% | +9q21, +11q13, +15q22, +19q13 |
| MM10 | 88% | +6q21, -13q, t(11;14) |
| MM11 | 79% | +1q21x2, -13q, t(4;14) |
| MM12 | 96% | +1q21, +4p16, +14q32, +17p13, +19q13 |
| MM13 | 70% | +15q22, +19q13x2 |
| MM14 | 67% | +9q21, +11q13, +15q22, -16q23, +19q13, |
| MM15 | 57% | +1q21, +4p16,+11q13, +15q22, +19q13 |
| MM16 | 87% | +1q21, +4p16, +9q21, +11q13, +15q22x2 , +16q23, +19q13 |
| MM17 | 97% | +9q21, +11q13, -13q, +15q22, +19q13 |
| MM18 | 89% | +1q21, -6q21, +9q21, -14q32, +15q22, -17p13 |
| MM19 | 59% | -13q, t(4;14) |
|  |  |  |
| MM20 | 0 | +1q21x2, -13, t(4;14) |
| MM21 | 0 | +1q21, -13q, t(14;16) |
| MM22 | 0 | normal |
| MM23 | 0 | t(11;14) |
| MM24 | 0 | +1q21 |
| MM25 | 0 | normal |
| K562 | 0 |  |
| U266 | 0 |  |
