# Supplementary material for: Deletion of Chromosomal Region 8p21 Confers Resistance to Bortezomib and Is Associated with Upregulated Decoy TRAIL Receptor Expression in Patients with Multiple Myeloma
Source: PLoS One. 2015 Sep 17;10(9):e0138248. doi: 10.1371/journal.pone.0138248 (PMC4574561; doi:10.1371/journal.pone.0138248)
Supplement: S4 Table — (DOCX) [file pone.0138248.s006.docx]

**S4 Table. Chromosomal abnormalities of patient cells used in TRAIL-R cell surface expression and apoptosis assays.**

Detailed cytogenetic characterization of the patient cohort used in the study. “+” indicates one additional signal (amplification) of the analyzed chromosomal region while x2 indicates 2 additional signals and “-” indicates loss of one signal. (nd: not done)

| Patient number | del(8)p(21) (%) | Other chromosomal abnormalities | 8cent/myc | TRAIL-R expression | Apoptosis |
| --- | --- | --- | --- | --- | --- |
| MM26 | 39 | +11q13 | 2c2myc (92%) |  | + |
| MM27 | 38 | +11q13 | 3c/3myc (28%) | + | + |
| MM28 | 98 | +1q21, - 6q21, -14q32, +15q22, -17p13 | 2c2myc (59%)  1c1myc (33%) | + | + |
| MM29 | 87 | +1q21, +4p16, +9q21 +11q13, +15q22, +16q23, +19q13 | 2c2myc (98%) |  | + |
| MM30 | 98 | +9q21 +11q13, - 13q, +15q22, +19q13 | 1c1myc (98%) | + |  |
| MM31 | 88 | +9q21, - 13q, +14q32, -16q23, -17p13 | 2c/2myc (95%) |  | + |
| MM32 | 98 | +1q21, t(14q32;?) | 2c/2myc (97%) | + | + |
| MM33 | 41 | +11q13, - 13, t(14q32;?) | 2c2myc (98%) | + |  |
| MM34 | 0 | +1q21, t(4;14) | 3myc (39%) | + | + |
| MM35 | 0 | +11q13 | nd | + | + |
| MM36 | 0 | t(11;14) | 2c3myc (32%) |  | + |
| MM37 | 0 | +11q13, -13q | 2c2myc (97%) |  | + |
| MM38 | 0 | +1q21,-4p16, -6q21,+9q21+11q13,+15q22, +19q13 | nd | + |  |
| MM39 | 0 | +1q21 | nd | + |  |
| MM40 | 0 | +1q21,-13,-14q32, -p17p13 | nd | + | + |
| MM41 | 0 | +11q13, - 13q, t(11;14) | nd |  | + |
| MM42 | 0 | +11q13 | nd |  | + |
| MM43 | 0 | +9q21 t(11;14), -13q, +15q22, +19q13 | nd |  | + |
| MM44 | 0 | +1q21, t(14:16) | nd | + | + |
| MM45 | 0 | +1q21, +9q21, -13q, +15q22 | nd | + | + |
| MM46 | 0 | normal | nd |  | + |
| MM47 | 0 | 1q21,-6q21, +9q21 +11q13, -13q, +14q32, +15q22, +19q13 | nd |  | + |
| MM48 | 0 | -4p16, -13q,-14q32, +15q22 | nd |  | + |
| MM49 | 0 | +1q21, +9q21 +11q13, +15q22, -16q23, +19q13 | nd |  | + |
