# Supplementary material for: Deletion of Chromosomal Region 8p21 Confers Resistance to Bortezomib and Is Associated with Upregulated Decoy TRAIL Receptor Expression in Patients with Multiple Myeloma
Source: PLoS One. 2015 Sep 17;10(9):e0138248. doi: 10.1371/journal.pone.0138248 (PMC4574561; doi:10.1371/journal.pone.0138248)
Supplement: S5 Table — (DOCX) [file pone.0138248.s007.docx]

| **Summary of TRAIL receptor expression before and after bortezomib treatment** | | | | | | |
| --- | --- | --- | --- | --- | --- | --- |
|  | **No del(8)(p21)** | | | **Del(8p)(21)** | | |
| BTZ | Mean | SD | Number | Mean | SD | Number |
| TRAIL-R1/isotype | | | | | | |
| - | 1.651429 | 0.513239 | 7 | 1.488 | 0.4017088 | 5 |
| + | 2.168572 | 0.737189 | 7 | 1.654 | 0.318873 | 5 |
| TRAIL-R2/isotype | | | | | | |
| - | 2.343125 | 1.481666 | 8 | 1.820 | 0.7927589 | 4 |
| + | 2.4875 | 1.458549 | 8 | 2.0275 | 0.7091485 | 4 |
| TRAIL-R3/isotype | | | | | | |
| - | 2.4225 | 0.7633339 | 8 | 3.298 | 1.619404 | 5 |
| + | 3.55375 | 1.788894 | 8 | 3.054 | 1.713952 | 5 |
| TRAIL-R4/isotype | | | | | | |
| - | 1.630 | 0.2836077 | 7 | 2.552 | 1.410149 | 5 |
| + | 1.858571 | 0.5459374 | 7 | 2.078 | 0.7253758 | 5 |

|  |  |
| --- | --- |
| **S5 Table. Summary of data in figures 3 and 4** |  |

| **Summary of the analysis of CD138^Bright^ cells with or without BTZ and/or TRAIL (%)** | | | | | | |
| --- | --- | --- | --- | --- | --- | --- |
|  | **No del(8)(p21)** | | | **del(8)(p21)** | | |
|  | mean | SD | Number | Mean | SD | Number |
| -BTZ/-TRAIL | 66.22 | 22.02 | 13 | 79.92 | 5.787 | 6 |
| +BTZ/-TRAIL | 47.53 | 20.71 | 13 | 65.32 | 19.48 | 6 |
| -BTZ/+TRAIL | 49.46 | 26.23 | 13 | 59.38 | 25.57 | 6 |
| +BTZ/+TRAIL | 28.15 | 17.98 | 13 | 42.93 | 27.89 | 6 |
|  | | | | | | |
| **Summary of the analysis of AnnexinV^-^/PI^-^ with or without BTZ and/or TRAIL (%)** | | | | | | |
|  | **No del(8)(p21)** | | | **del(8)(p21**) | | |
|  | mean | SD | Number | Mean | SD | Number |
| -BTZ/-TRAIL | 76.80 | 11.41 | 13 | 78.33 | 14.96 | 6 |
| +BTZ/-TRAIL | 57.36 | 22.48 | 13 | 75.08 | 13.82 | 6 |
| -BTZ/+TRAIL | 63.07 | 18.12 | 13 | 70.93 | 17.74 | 6 |
| +BTZ/+TRAIL | 40.88 | 19.73 | 13 | 65.08 | 23.77 | 6 |
